# Expansion of Surveillance for Vaccine-preventable Diseases: Building on the Global Polio Laboratory Network and the Global Measles and Rubella Laboratory Network Platforms

**DOI:** 10.1093/infdis/jix077

**Published:** 2017-06-30

**Authors:** Mick N. Mulders, Fatima Serhan, James L. Goodson, Joseph Icenogle, Barbara W. Johnson, Paul A. Rota

**Affiliations:** 1 Expanded Program on Immunization, World Health Organization, Geneva, Switzerland; and; 2 Centers for Disease Control and Prevention, Atlanta, Georgia

**Keywords:** measles, rubella, polio, yellow fever, Japanese encephalitis, rotavirus, invasive bacterial disease, laboratory network, surveillance.

## Abstract

Laboratory networks were established to provide accurate and timely laboratory confirmation of infections, an essential component of disease surveillance systems. The World Health Organization (WHO) coordinates global laboratory surveillance of vaccine-preventable diseases (VPDs), including polio, measles and rubella, yellow fever, Japanese encephalitis, rotavirus, and invasive bacterial diseases. In addition to providing high-quality laboratory surveillance data to help guide disease control, elimination, and eradication programs, these global networks provide capacity-building and an infrastructure for public health laboratories. There are major challenges with sustaining and expanding the global laboratory surveillance capacity: limited resources and the need for expansion to meet programmatic goals. Here, we describe the WHO-coordinated laboratory networks supporting VPD surveillance and present a plan for the further development of these networks.

## Summary

Laboratory networks provide accurate and timely laboratory confirmation of infections, an essential component of disease surveillance. The World Health Organization coordinates global laboratory surveillance for many vaccine-preventable diseases. This report describes these laboratory networks and the challenges in sustaining and expanding global laboratory surveillance capacity.

Laboratory-supported surveillance is a critical component for strategies to control, eliminate, and eradicate infectious diseases, including vaccine-preventable diseases (VPDs). Surveillance is based on principles of rapid case detection, reporting, and epidemiologic and laboratory investigation. In settings with established regional goals for disease elimination or eradication, high-quality, case-based surveillance (including laboratory confirmation) is required to guide program activities toward achieving milestones, and to verify disease elimination or eradication.

The World Health Organization (WHO) and Member States coordinate global laboratory networks to support surveillance for selected VPDs, including polio, measles, rubella, yellow fever (YF), Japanese encephalitis (JE), rotavirus, influenza, tuberculosis, and invasive bacterial disease (IBD) [1]. Here, we describe the VPD laboratory surveillance networks for polio, measles, rubella, YF, JE, rotavirus, and IBD. This report focuses on the challenges faced in sustaining and expanding global laboratory surveillance capacity, and presents a plan for further development as part of the global polio transition plan. The WHO’s Global Influenza Surveillance and Response System and TB Supranational Reference Laboratory Network were not built on the polio and measles–rubella platforms, and are too complex to describe in this manuscript.

## GLOBAL LABORATORY NETWORKS FOR VPD SURVEILLANCE

### Polio

Following the adoption of a global goal to eradicate polio by the World Health Assembly (WHA) in 1988, the WHO developed a global network of laboratories to ensure high-quality laboratory diagnosis of suspect cases of poliomyelitis [2]. The Global Polio Laboratory Network (GPLN) [3], built in the late 1980s with a pyramidal 3-tiered structure design, includes 146 laboratories with complementary capacities. The tiered structure includes national laboratories (NLs) linked to a Regional Reference Laboratory (RRL), and Global Specialized Laboratories (GSLs). RRLs provide confirmatory testing and training for the NLs. The GSLs work closely with the WHO to develop and improve standardized laboratory diagnostic methods for poliovirus isolation, molecular strain characterization, quality assurance, and database management. Fully integrated with case-based surveillance for acute, flaccid paralysis cases, the GPLN provides poliovirus case confirmation, intratypic differentiation between vaccine-derived and wild-type poliovirus strains, and molecular epidemiological data for guiding immunization activities. This global laboratory capacity requires considerable investments in laboratory infrastructure, equipment, supplies, reagents, quality assurance, staffing, and training, often in resource-limited settings. The GPLN helped establish standard surveillance performance indicators, as well as a laboratory quality control and accreditation programs. The GPLN has served as a model for the development of other global laboratory networks making contributions that will continue long after the polio eradication goal is achieved.

### Measles and Rubella

In 2012, the WHA endorsed the Global Vaccine Action Plan and its objective to eliminate measles and rubella in 5 of the 6 WHO regions by 2020. As of September 2013, countries in all 6 WHO regions had adopted measles elimination goals, and in 3 regions, additional goals for the elimination of rubella and congenital rubella syndrome (CRS) had been adopted [4]. Case-based surveillance is a key strategy for monitoring transmission, informing vaccination activities, and verifying elimination. The Global Measles and Rubella Laboratory Network (GMRLN) was established to provide high-quality, standardized testing to support case-based surveillance [5–7]. Development of the GMRLN started in 2000 using a multi-tiered structure similar to the design of the GPLN. As of January 2016, the GMRLN has 703 laboratories in 165 countries serving 191 countries, including 506 subnational, 180 NLs, 14 RRLs, and 3 GSLs, compared to the 146 laboratories comprising GPLN. Similarly, the GMRLN has a Global Laboratory Coordinator based at the WHO headquarters in Geneva, and each WHO region has a Regional Laboratory Coordinator. NLs and subnational laboratories are closely linked with the national immunization program and perform laboratory testing for case confirmation. The national laboratories are supported by the RRLs that serve as regional centers of excellence, provide quality-control testing, proficiency testing, training, and genetic characterization of circulating viruses. The GSLs contribute to the standardization of procedures and protocols, development and validation of novel methods, and support-capacity building through training. Similar to the GPLN, the GMRLN supports case-based surveillance that aims for global coverage. The GMRLN supports surveillance in 191 Member States and provides an opportunity for integrated surveillance for other vaccine-preventable diseases, including rotavirus, IBD, YF, and JE [5, 8].

The capacity of the GMRLN continues to provide laboratory support to all countries with detection of measles- or rubella-specific immunoglobulin type M (IgM) by enzyme immune assay as the most widely used method for case confirmation (Table 1). In 2015, among the 160 countries that reported case-based surveillance data, 143 105 serum specimens were received by the GMRLN for testing (Table 1). Of these specimens, 131 513 (92%) were tested for measles IgM (34 459 [26%] positive), and 109267 (76%) were also tested for rubella IgM (13142 [12%] positive) [9].

**Figure 1.**
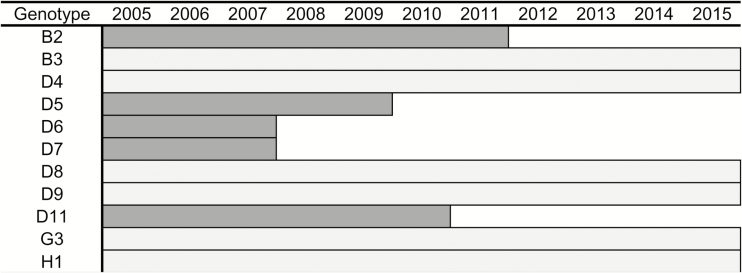
Measles virus genotypes detected during 2005–2015 by the Global Measles and Rubella Laboratory Network (GMRLN). Dark gray bars indicate genotypes detected in 2005 that are likely no longer circulating; light gray bars indicate genotypes currently circulating.

To support virologic surveillance, the WHO established standard protocols for monitoring global genotype distribution and tracking transmission of measles and rubella viruses [10–12]. Genotype data are reported to the WHO sequence databases, the Measles Nucleotide Surveillance (MeaNS) database, and the Rubella Nucleotide Surveillance (RubeNS) database [13]. During 2010–2015, 25831 measles virus sequences were submitted to MeaNS and 855 rubella virus sequences were submitted to RubeNS (Table 1). To monitor the global transmission patterns of defined lineages of measles virus, a procedure was introduced to MeaNS that designates eligible measles sequences from contemporary outbreak strains as “named strains” [10]. Of the 24 recognized measles virus genotypes, 11 were detected in 2005, and of those, 6 were detected in 2015 (Figure 1).

**Table 1.** Summary of Serologic and Molecular Testing by the Global Measles and Rubella Laboratory Network (GMRLN), 2010–2015

**Year**	**2010**	**2011**	**2012**	**2013**	**2014**	**2015**
	Number of Suspected Cases with Serum Specimens Tested for Measles-specific and/or Rubella-specific IgM^a^					
JRF	171170	152810	148177	197469	258339	226004
Monthly^b^	64864	85953	122719	160611	161115	131513
Percent serum samples testing positive for measles or rubella IgM^c^						
Measles	NA	NA	32	31	37	26
Rubella	NA	NA	17	10	11	12
Number of sequences submitted to the GMRLN databases^d^						
Measles	4329	5817	2911	2521	7368	8691
Rubella	67	143	112	39	148	346

Abbreviations: IgM, immunoglobulin M; JRF, joint reporting form; MeaNS, Measles Nucleotide Surveillance database; NA, data not available; RubeNS, Rubella Nucleotide Surveillance database.

^a^Testing schemes for detection of measles and rubella IgM vary by region and country.

^b^Monthly reporting.

^c^Samples that test negative for measles IgM are tested for rubella IgM.

^d^Data reported to MeaNS (www.who-measles.org) and RubeNS (www.who-rubella.org) as of 15 June 2016.

Following the experience of the GPLN, an annual accreditation and proficiency testing program for the GMRLN ensures high-quality laboratory testing (Table 1). Since 2010, approximately 95% of participating laboratories pass annual accreditation. The number of laboratories that participated in the molecular proficiency testing program increased from 22 in 2014 to 50 in 2016; all laboratories passed the molecular proficiency test in 2014 and 97% of the laboratories passed in 2015 (the results for 2016 are still pending). The few laboratories that fail annual accreditation or the serologic and molecular proficiency tests are contacted by the Regional Laboratory Coordinator to devise a plan for expedited remedial action. The core performance indicators to monitor the quality of measles and rubella laboratory-based surveillance are timeliness of reporting laboratory results, reporting rate of discarded nonmeasles and nonrubella cases, proportion of cases with adequate clinical specimen collected and tested in a proficient laboratory, and proportion of laboratory-confirmed chains of transmission, with specimens adequate for detecting measles or rubella virus collected and tested in an accredited laboratory. Most laboratories (>95%) meet the target of ≥80% for these 5 indicators.

In 2013, the Strategic Advisory Group of Experts (SAGE) on Immunization recommended a framework for verifying the elimination of measles and rubella that included 3 criteria for the verification of measles or rubella elimination [14]. Two of these criteria—the presence of a high-quality surveillance system and genotyping evidence that supports the interruption of transmission—rely heavily on data provided by the GMRLN laboratories. Thus far, the Region of the Americas (AMR) is the only region to have certified measles and rubella elimination [15, 16].

Surveillance data are used to help monitor rubella virus transmission and to prevent CRS. An approach to verifying the elimination of CRS in the AMR was published in 2011 [17]. In the AMR, it was necessary to complement routine reporting systems with a retrospective search for suspected CRS cases, using various sources of information for at least the 3 years preceding the certification date. Globally, overall guidance for CRS surveillance has been provided [14], and specific recommendations for CRS surveillance indicators are now being finalized. Such recommended-specific CRS surveillance indicators will likely require timely case identification and investigation, including collection of appropriately timed specimens for laboratory confirmation and investigation. The GMRLN laboratories can perform the laboratory testing needed to confirm cases of suspect CRS, and the GMRLN is expanding regional training for the detection and monitoring of CRS cases by engaging both laboratory scientists and epidemiologists.

### Yellow Fever

Yellow fever virus is an arthropod-borne virus with both human and nonhuman primate transmission cycles, which causes sporadic outbreaks in Africa and the Americas. The recognition of YF cases in the early stages of an outbreak is difficult because the differential diagnosis considers several diseases, including malaria, viral hepatitis, dengue, leptospirosis, or other hemorrhagic fevers. However, 1 laboratory-confirmed YF case may initiate an outbreak investigation, necessitating a response, which may include mass vaccination campaigns. A definitive diagnosis of YF infection cannot be made based solely on clinical impressions, and laboratory confirmation is necessary for final case classification [18, 19]. The WHO has established a global tiered YF laboratory network (GYFLN) comprising more than 40 laboratories in the AMR and the African Region (AFR), predominantly within existing GPLN and GMRLN laboratories, which capitalize on investments by global partners to develop those networks. The terms of reference of the GSLs, RRLs, and NLs are similar to the other networks. High-quality commercial diagnostic kits for IgM detection are not available, and laboratories must rely on IgM capture assays produced by GSLs at the CDC and Pasteur Institute of Dakar for case confirmation. IgM detection remains the primary diagnostic test in the NLs with limited technical capacity. Confirmation of positive results by the RRL is an essential component of the GYFLN because of the cross-reactivity of YF virus–specific IgM antibodies with IgM antibodies elicited against cocirculating flaviviruses. Following capacity-building by partner organizations for other VPDs, some laboratories in the GYFLN are now using molecular techniques for case confirmation. Improved diagnostic assays for YF in resource-limited settings is a critical need for the GYFLN.

### Japanese Encephalitis

A similar approach has been taken to establish and coordinate the laboratory network for Japanese encephalitis (JELN), which is a major cause of childhood mortality and morbidity in countries of South-East Asia Region (SEAR) and Western Pacific Region (WPR). It is the most important cause of arboviral encephalitis globally. JE is a zoonotic disease, transmitted between mosquito vectors and nonhuman vertebrate hosts (primarily birds and pigs). Vaccination is the only effective protection for humans living in areas where the JE virus is circulating. Approximately 3 billion people live in JE-endemic regions, and JE causes at least 50000 acute encephalitis syndrome (AES) cases with an estimated 10000 deaths annually [20]. The JE vaccine was WHO-prequalified in 2013.

Introduction of the JE vaccine will reduce the number of JE cases, but it will also increase the need for enhanced surveillance to determine the disease burden and trends, substantiate the need for vaccination, monitor the impact of vaccination programs, and detect outbreaks [21]. Laboratory confirmation of JE infection is essential for accurate surveillance, to determine the disease burden and trends, substantiate the need for vaccination, monitor impact of vaccination programs, and to detect outbreaks. The JELN, a tiered laboratory network comprising 25 laboratories, has been established in the SEAR (n = 15) and WPR (n = 10), with an additional 10 subnational JE laboratories added to the Chinese network in 2013, building on the existing GPLN and GMRLN. JE testing is based on the detection of IgM antibodies in clinical specimens, particularly in cerebrospinal fluid. As the proportion of subclinical infections during a JE outbreak is very high (≥90%), testing for IgM in serum samples needs to be interpreted carefully, as other pathogens may be the cause of AES. Like the YF virus, the JE virus is a flavivirus, and cross-reactivity of JE virus–specific IgM with cocirculating flaviviruses (eg, dengue) can confound a diagnosis. Therefore, standardized differential diagnostic testing has been an essential component for the JELN. Furthermore, in contrast to IgM for measles or rubella, JE virus–specific IgM antibodies may not de detectable if acute serum samples are collected within 7 days of disease onset; a second serum sample may be required. Coordination, quality assurance, and accreditation for both the GYFLN and JELN have been implemented following the models established by the GPLN and GMRLN [22]. Annual proficiency testing was established in 2010. Many NLs participated for the first time in 2015 and 86% of them passed.

### Rotavirus

Rotavirus is the most common cause of severe diarrhea among children under 5 years of age [23, 24]. In 2009, the WHO recommended that countries (particularly those with high childhood mortality from diarrhea) introduce rotavirus vaccines into their national immunization system [25]. Surveillance data on laboratory-confirmed rotavirus cases were important in order to allow countries to make informed decisions on vaccine introduction. By the end of 2014, more than 70 countries had introduced rotavirus vaccine into their routine childhood immunization program. Following the introduction of rotavirus vaccines, it is important to monitor the impact of vaccination in reducing rotavirus morbidity and mortality, evaluate vaccine effectiveness, detect the emergence of rotaviruses that are not prevented by vaccine-induced antibodies, and monitor the safety of rotavirus vaccines [26]. To monitor trends of severe rotavirus disease, sentinel surveillance (including laboratory testing) was established at health-care facilities.

**Figure 2.**
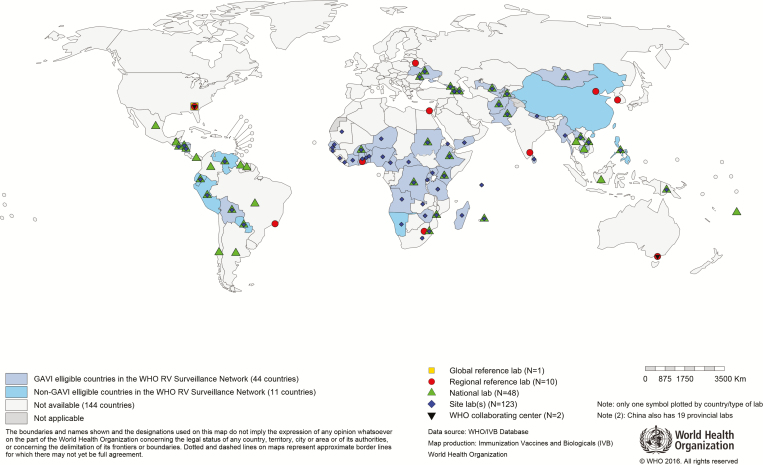
Map showing the countries that contributed data to the Global WHO-coordinated GRLN data in 2015. The global rotavirus laboratory network is an integral component of surveillance, and every country that reported surveillance data to WHO has at least 1 sentinel hospital site laboratory that performed enzyme immune assay rotavirus testing. All national laboratories highlighted have genotyping capacities.

Genotype surveillance is important to monitor possible shifts in rotavirus genotypes [27]. In 2008, WHO established the Global Rotavirus Laboratory Network (GRLN) based on previously existing regional networks [28]. The GRLN supports laboratory testing for stool samples collected from hospitalized children with severe diarrhea (Figure 2). The function and structure are similar to those of the GPLN and GMRLN, with GSLs and RRLs providing technical support to NLs and hospital laboratories. The initial testing is performed at the sentinel hospital level with WHO-recommended, commercial enzyme immunoassay (EIA) kits [29]. Genotyping of rotavirus-positive samples is performed at the NL or RRL. In 2015, the laboratory network tested 45240 of 49078 (92.18%) stool samples collected from diarrheal cases. Of those samples, 12429 (27.47%) were tested by EIA and were positive for rotavirus, and 3238 genotypes from 25 countries were obtained.

The GRLN has adopted approaches similar to those of the GPLN and GMRLN to confirm and improve the accuracy of collected data. The GRLN monitors the laboratory performance through site visits, data review and analysis, external quality control program for confirmatory testing, and annual external quality assurance (EQA) using proficiency test panels. In 2015, 113 (97%) of 116 laboratories that participated in EQA passed the EIA testing, and 49 (91%) of 54 labs that had genotyping capacities passed the molecular quality control. Since 2013, the GRLN has been used as a platform for surveillance for other diarrheal pathogens (including the use of molecular diagnostic technologies) for the simultaneous detection of more than 20 enteric pathogens, including bacteria, viruses, and protozoa.

### Invasive Bacterial Disease

The WHO and partners coordinate a global sentinel surveillance network in selected hospitals for vaccine-preventable IBD that was brought together from preexisting regional IBD networks in 2008. In 2015, the global IBD laboratory network included 117 sentinel hospitals, 20 NLs, 9 RRLs, and the CDC Global Reference Laboratory. The objectives of this network are to gather standardized data on children under 5 years of age suspected to have contracted invasive, severe infection caused by *Streptococcus pneumoniae*, *Haemophilus influenzae*, and *Neisseria meningitidis*. These data are used by policy-makers to inform evidence-based decisions on vaccine introduction and to assess the effectiveness of vaccine introduction by monitoring disease trends and serotype/serogroup distribution before and after vaccine introduction [30]. The IBD laboratory network has followed similar approaches of the aforementioned laboratory networks. The complexity of bacteriologic testing and the limited laboratory capacities for diagnosis of bacterial meningitis in countries were significant challenges in expanding the IBD network. However, the IBD network has made great progress in enhancing the capacity to test for bacteriologic pathogens, and it is the first established global laboratory network for bacteriology, providing high-quality data for IBD surveillance [31].

Cerebrospinal fluid specimens are collected from suspected meningitis cases and tested in a laboratory (eg, gram stain, bacterial culture, and, where available, a rapid diagnostic test based on immunochromatography or latex agglutination). Culture is performed on clinical specimens collected from suspected pneumonia and sepsis cases. A system of specimen referral between the sentinel hospital laboratories and the NL or RRL has been established to identify the pathogens from clinical samples. The IBD network has established quality-assurance and quality-control systems for laboratory testing. In 2015, 90 of the 98 IBD network laboratories that participated in the EQA program passed.

## LESSONS LEARNED FROM VPD LABORATORY NETWORKS

A continuing and significant challenge to all of the WHO-coordinated laboratory networks is the long-standing shortage of human and financial resources. Global donors, as well as national governments, often earmark funds for interventions such as vaccines or injection devices, but fail to recognize the critical role of surveillance to detect diseases and monitor vaccine impacts. Surveillance laboratory networks require sustained investments to support operations that are often in resource-limited and in logistically challenging settings. To some extent, integration of efforts across laboratory networks is possible; therefore, resources that support one network may also have a beneficial effect on other networks. For example, the GMRLN was largely built on (and is still partially supported by) the existing GPLN, and, in turn, the YF and JE surveillance networks were built onto the GMRLN. Of serious concern and a major risk to global VPD surveillance is the imminent diminishing of resources currently supporting polio eradication. There is an urgent need to communicate to donors the important role that disease surveillance plays, and to mitigate the threat of resources flatlining or decreasing, particularly once polio eradication is achieved.

Another significant challenge is the needed expansion of the networks to meet increasing programmatic demands for high-quality surveillance data. Within the GMRLN network (particularly in SEAR), there is an immediate challenge to expand as new laboratories are designated in India, Thailand, Myanmar, Nepal, and Indonesia. Accommodating this expansion will require additional resources.

Maintaining a well-trained laboratory workforce is often challenging because of staff turnover, changing programmatic demands, and the introduction of new or modified methods. All of the laboratory networks have ongoing training activities through onsite training, intercountry workshops and meetings, laboratory visits and, more recently, electronic media (such as videos and webinars). The GPLN has developed a training program (including standard laboratory practices and procedures and biosafety videos) that is being used by all the laboratory networks. Training needs contribute significantly to the workload and resource requirements of the RRLs, GSLs, and Regional Laboratory Coordinators, and this workload is likely to increase as networks expand.

A major logistic challenge is maintaining an uninterrupted availability of supplies and reagents needed to perform the transportation and testing of specimens. As laboratory networks expand, the number of specimens collected and shipped, laboratory tests performed, and associated logistic issues increase. Local specimen transport requires maintenance of a reliable infrastructure for the reverse cold chain. International shipment of reagents, supplies, and clinical samples is becoming more expensive and time consuming because of more stringent regulations. Logistical issues are especially challenging during outbreaks (where there is a need for surge capacity for laboratory staff and supplies and reagents), and close coordination is required to avoid shortages and to ensure that data are collected to guide outbreak-response activities in a timely manner.

Responding to the differing programmatic requirements of case-based, syndromic, and sentinel surveillance will be a new challenge as network laboratories move toward more integrated disease surveillance. The various specimen types have different reverse cold chain requirements, and the testing will require a wide range of techniques. Integrated testing for viral and bacterial pathogens may require a substantial amount of additional resources and training [32]. Finally, standardized testing methods must be available for all VPDs.

The laboratory networks could not function properly without coordination at the global, regional, and national levels of participating laboratories. Coordination is essential and allows for the maintenance of standard performance indicators, quality assurance and accreditation programs, standardized testing methods and data collection, and effective interfacing with the epidemiological units of national programs. Close collaboration to strengthen the networks creates esprit de corps for building long-lasting partnerships to achieve public health goals. However, maintaining the financial support for global and regional coordination is an ongoing challenge.

## FUTURE OF VPD SURVEILLANCE NETWORKS IN A POSTPOLIO ERADICATION WORLD

The development of the GPLN demonstrated the great value of having globally coordinated laboratory surveillance, and provided infrastructure for public health laboratories that facilitated the development of the GMRLN and other disease surveillance laboratory networks. Laboratories in the GMRLN have benefited by the infrastructure provided by the GPLN, including the establishment of dedicated cell culture and polymerase chain reaction (PCR) facilities, including the availability of key equipment such as thermocyclers and automated sequencers, which are readily available for other disease surveillance activities. Because IgM detection is still the method of choice for the confirmation of measles and rubella cases, the GMRLN has helped to establish serologic testing for other viral diseases in many laboratories. This capacity for serologic testing is now being used by laboratories in the AMR, AFR, SEAR, and WPR to offer serologic testing for YF and JE. Also, many GMRLN laboratories have established molecular testing, including genotyping—techniques that will prove useful for detection of other pathogens.

The GPLN laboratories developed a work culture that valued timely and accurate testing with a strong foundation of capacity-building, quality control, data management, and biosafety and biosecurity, and this culture is shared by the other WHO-coordinated laboratory networks. The laboratory accreditation program initiated by the GPLN was adapted for use by the GMRLN and provides a basis for other assessment programs. In addition, these networks have built capacity though continuous training efforts. The GPLN and GMRLN networks have developed strong programs for referral of samples for confirmation, and both have developed external quality assurance programs through mandatory proficiency panels to assess the performance of serologic and molecular testing.

To enhance laboratory-based surveillance, the global laboratory networks are exploring new technologies to replace virus isolation for the identification of pathogens and serologic methods (such as the detection of pathogen-specific IgM) for case confirmation. These technologies are often based on common platforms such as real-time reverse transcriptase–PCR, next-generation sequencing, and high-throughput serologic testing. For example, there is an increasing use of PCR for laboratory confirmation. The network laboratories can take advantage of these common platforms and develop assays that are amenable to supporting surveillance for many viral and bacterial diseases.

To secure the investments made by the GPLN, we now have the opportunity to transition the resources targeted for the GPLN to further expand the laboratory networks and to develop an integrated approach that can support global surveillance for VPDs, including polio. The key objective of the transition is to maintain laboratory support for high-quality, case-based, and syndromic surveillance systems that meet performance indicators and can provide high-quality surveillance data needed to verify disease control and progress toward elimination. Efforts are underway to develop next-generation information systems to optimize the use of data for immunization program monitoring and VPD surveillance. Utilization of GPLN assets and experience to help achieve existing goals for other programs is a cost-effective way to transition the Global Polio Eradication Initiative, while developing integrated VPD surveillance to maintain needed polio surveillance capacity.

## Notes

***Acknowledgments.*** The authors thank Jon Gentsch, David A. Featherstone, and Adam Cohen for helpful comments on this manuscript. The authors also acknowledge the tremendous contributions of the Regional Laboratory Coordinators for the GPLN, GMRLN, GRLN, GYFLN, and JELN and IBD Laboratory network.

***Supplement sponsorship.*** This work is part of a supplement coordinated by the Task Force for Global Health with funding provided by The Bill and Melinda Gates Foundation and the Centers for Disease Control and Prevention.

***Potential conflicts of interest.*** All authors: No reported conflicts. All authors have submitted the ICMJE Form for Disclosure of Potential Conflicts of Interest. Conflicts that the editors consider relevant to the content of the manuscript have been disclosed.

## References

[1] Diop OM, Kew O, de Gourville E, Pallansch MA The global polio laboratory network as a platform for the viral vaccine preventable and emerging diseases laboratory networks. J Infect Dis 2017; 216 (suppl 1):S299–307. 10.1093/infdis/jix092 PMC5853949 28838192

[2] Hull BP, Dowdle WR Poliovirus surveillance: building the global Polio Laboratory Network. J Infect Dis 1997; 175 Suppl 1:S113–6. 9203702 10.1093/infdis/175.supplement_1.s113

[3] de Gourville E, Duintjer Tebbens RJ, Sangrujee N, Pallansch MA, Thompson KM Global surveillance and the value of information: the case of the global polio laboratory network. Risk Anal 2006; 26:1557–69. 17184397 10.1111/j.1539-6924.2006.00845.x

[4] Anonymous. Global vaccine action plan. decade of vaccine collaboration. Vaccine 2013; 31 Suppl 2:B5–31. 23734366 10.1016/j.vaccine.2013.02.015

[5] Mulders M, Rota P, Icenogle J Global measles and rubella laboratory network support for elimination goals, 2010–2015. MMWR Morb Mortal Wkly Rep 2016; 65:438–42. 27148917 10.15585/mmwr.mm6517a3

[6] Featherstone D, Brown D, Sanders R Development of the global measles laboratory network. J Infect Dis 2003; 187 Suppl 1:S264–9. 12721924 10.1086/368054

[7] Featherstone DA, Rota PA, Icenogle J Expansion of the global measles and rubella laboratory network 2005–09. J Infect Dis 2011; 204 Suppl 1:S491–8. 21666205 10.1093/infdis/jir107

[8] CDC. Expanding poliomyelitis and measles surveillance networks to establish surveillance for acute meningitis and encephalitis syndromes—Bangladesh, China, and India, 2006–2008. MMWR Morb Mortal Wkly Rep 2012; 61:1008–11. 23235298

[9] WHO. Measles Surveillance Data http://www.who.int/immunization/monitoring_surveillance/burden/vpd/surveillance_type/active/measles_monthlydata/en/ Accessed 2 April 2017.

[10] WHO. Genetic diversity of wild-type measles viruses and the global measles nucleotide surveillance database (MeaNS). Wkly Epidemiol Rec 2015; 90:373–80. 26211016

[11] WHO. Measles virus nomenclature update: 2012. Wkly Epidemiol Rec 2012; 87:73–81. 22462199

[12] WHO. Expanded Programme on Immunization (EPI). Standardization of the nomenclature for describing the genetic characteristics of wild-type measles viruses. Wkly Epidemiol Rec 1998; 73:265–9. 9745371

[13] Rota PA, Brown KE, Hübschen JM Improving global virologic surveillance for measles and rubella. J Infect Dis 2011; 204 Suppl 1:S506–13. 21666207 10.1093/infdis/jir117

[14] Framework for verifying elimination of measles and rubella. Wkly Epidemiol Rec **2013**; 88:89–99 . 23540051

[15] PAHO. Americas region is declared the world’s first to eliminate rubella http://www.paho.org/hq/index.php?option=com_content&view=article&id=10798%3A2015-americas-free-of-rubella&catid=740%3Apress-releases&Itemid=1926&lang=pt Accessed 2 April 2017.

[16] PAHO. Region of the Americas is declared free of measles http://www.paho.org/hq/index.php?option=com_content&view=article&id=12528&Itemid=1926&lang=en Accessed 2 April 2017.

[17] Castillo-Solórzano C, Reef SE, Morice A Guidelines for the documentation and verification of measles, rubella, and congenital rubella syndrome elimination in the region of the Americas. J Infect Dis 2011; 204 Suppl 2:S683–9. 21954267 10.1093/infdis/jir471

[18] WHO. Manual for the monitoring of yellow fever virus infection http://www.who.int/immunization/monitoring_surveillance/burden/laboratory/Manual_YF.pdf?ua=1 Accessed 2 April 2017.

[19] WHO. Yellow fever laboratory diagnostic testing in Africa Interim guidance http://apps.who.int/iris/bitstream/10665/246226/1/WHO-OHE-YF-LAB-16.1-eng.pdf?ua=1 Accessed 3 April 2017.

[20] Campbell GL, Hills SL, Fischer M Estimated global incidence of Japanese encephalitis: a systematic review. Bull World Health Organ 2011; 89:766–74, 774A–774E. 22084515 10.2471/BLT.10.085233 PMC3209971

[21] Hills S, Martin R, Marfin A, Fischer M Control of Japanese encephalitis in Asia: the time is now. Expert Rev Anti Infect Ther 2014; 12:901–4. 24927959 10.1586/14787210.2014.929498 PMC4594829

[22] WHO. Manual for the Laboratory Diagnosis of Japanese Encephalitis Virus Infection http://www.wpro.who.int/immunization/documents/Manual_lab_diagnosis_JE.pdf Accessed 2 April 2017.

[23] CDC. Rotavirus surveillance—worldwide, 2001–2008. MMWR Morb Mortal Wkly Rep 2008; 57:1255–7. 19023263

[24] Patel MM, Glass R, Desai R, Tate JE, Parashar UD Fulfilling the promise of rotavirus vaccines: how far have we come since licensure? Lancet Infect Dis 2012; 12:561–70. 22742639 10.1016/S1473-3099(12)70029-4

[25] WHO. Generic protocols for (i) hospital-based surveillance to estimate the burden of rotavirus gastroenteritis in children and (ii) a community-based survey on utilization of health care services for gastroenteritis in children: field test version http://apps.who.int/iris/bitstream/10665/67743/1/WHO_V-B_02.15_eng.pdf Accessed 3 April 2017.

[26] WHO. Rotavirus vaccines WHO position paper: January 2013—Recommendations. Vaccine 2013; 31:6170–1. 23746456 10.1016/j.vaccine.2013.05.037

[27] Tate JE, Burton AH, Boschi-Pinto C 2008 estimate of worldwide rotavirus-associated mortality in children younger than 5 years before the introduction of universal rotavirus vaccination programmes: a systematic review and meta-analysis. Lancet Infect Dis 2012; 12:136–41. 22030330 10.1016/S1473-3099(11)70253-5

[28] WHO. Building rotavirus laboratory capacity to support the Global Rotavirus Surveillance Network. Wkly Epidemiol Rec 2013; 88:217–23. 23757795

[29] WHO. Manual of rotavirus detection and characterization methods http://apps.who.int/iris/bitstream/10665/70122/1/WHO_IVB_08.17_eng.pdf Accessed 3 April 2017.

[30] Levine OS, Cherian T, Hajjeh R, Knoll MD Progress and future challenges in coordinated surveillance and detection of pneumococcal and Hib disease in developing countries. Clin Infect Dis 2009; 48 Suppl 2:S33–6. 19191617 10.1086/596479

[31] WHO. WHO Global Invasive Bacterial Vaccine Preventable Disease and Rotavirus Surveillance Network https://goo.gl/96tF4i Accessed 2 April 2017.

[32] Cavallaro KF, Sandhu HS, Hyde TB Expansion of syndromic vaccine preventable disease surveillance to include bacterial meningitis and Japanese encephalitis: evaluation of adapting polio and measles laboratory networks in Bangladesh, China and India, 2007–2008. Vaccine 2015; 33:1168–75. 25597940 10.1016/j.vaccine.2015.01.004 PMC4830482

